# In Search of the Driving Factor for the Microwave Curing of Epoxy Adhesives and for the Protection of the Base Substrate against Thermal Damage

**DOI:** 10.3390/molecules26082240

**Published:** 2021-04-13

**Authors:** Satoshi Horikoshi, Yuhei Arai, Nick Serpone

**Affiliations:** 1 Department of Materials and Life Sciences, Faculty of Science and Technology, Sophia University, 7-1 Kioicho, Chiyodaku, Tokyo 102-8554, Japan; y-arai-wp8@eagle.sophia.ac.jp; 2 PhotoGreen Laboratory, Dipartimento di Chimica, Università degli Studi di Pavia, Via Taramelli 12, 27100 Pavia, Italy; nick.serpone@unipv.it

**Keywords:** microwave, epoxy adhesive, thermal damage, microwave-assisted curing, penetration depth, electric field, magnetic field

## Abstract

This study used controlled microwaves to elucidate the response of adhesive components to microwaves and examined the advantages of microwave radiation in curing epoxy adhesives. Curing of adhesives with microwaves proceeded very rapidly, even though each component of the adhesive was not efficiently heated by the microwaves. The reason the adhesive cured rapidly is that microwave heating was enhanced by the electrically charged (ionic) intermediates produced by the curing reaction. In contrast, the cured adhesive displayed lower microwave absorption and lower heating efficiency, suggesting that the cured adhesive stopped heating even if it continued to be exposed to microwaves. This is a definite advantage in the curing of adhesives with microwaves, as, for example, adhesives dropped onto polystyrene could be cured using microwave heating without degrading the polystyrene base substrate.

## 1. Introduction

Automobiles are generally fabricated entirely with metallic components, which add undue weight and thus cause reduced fuel efficiency. Accordingly, car makers are now focusing on multi-material components to decrease the weight and thus achieve greater fuel efficiency [1]. Welding and bolting are typically used for an all-metal car body; however, welding and bolting cannot be used to join/connect dissimilar materials such as metal with glass, metal with plastics, and metal with carbon-fiber reinforced plastics. Consequently, adhesives have been developed and used to combine the various assorted car components. Another technology where adhesive bonding is used is in the fabrication of smartphones, the number of which exceeded 1.2 billion in 2020 alone and is likely to witness a rapid increase in the near future. With 5G communications coming online, a metal housing for 5G high-speed, high-frequency, printed circuit boards has become impracticable [2]. Thus, the use of various adhesives to bond dissimilar materials has become the norm. It is expected that additional multi-materials and composite materials will be developed in the near future [3], which will witness further expansion of adhesives to assemble various products.

Adhesives that cure in a short time tend to be poorly stable during storage, while adhesives that are stable during storage have some problems with curing speed and present both advantages and disadvantages. Therefore, mainstream adhesives consist of a plurality of adhesive components that are mixed immediately before use, followed by heat-curing using various heat sources or otherwise using ultraviolet (UV) energy to drive the curing process, followed by the hardening event. However, there are some difficulties with thermosetting and UV curing. In addition, in the case of heat-assisted curing, bonding the components in electronic materials (e.g., circuit boards) and curing adhesives in heat-sensitive materials are neither desirable nor recommendable as they might damage the material components. A disadvantage of UV curing is that the UV rays do not reach all parts of the assembly. Consequently, a new energy source is required and desirable to drive the curing reaction.

Microwave radiation is an energy source that also consists of electromagnetic waves, as UV rays are, and since only those materials that absorb the microwave energy undergo self-heating, selective microwave heating is then possible [4]. Moreover, microwave selective heating makes it possible to heat/cure the adhesive components while simultaneously allow for cooling by external means [5]. Although microwaves are also electromagnetic waves (not light waves but radio waves), unlike UV rays, however, there are no components that microwaves cannot reach.

Several studies have examined microwave radiation as a potential heat source to cure epoxy adhesives. They have shown that microwave-assisted curing provided mechanical characteristics of the adhesive similar to those from curing using conventional heat transfer [6,7]. Indeed, equivalent or better mechanical characteristics were obtained by microwave curing, in comparison to those obtained by conventional thermal curing, with microwave curing providing shorter curing times and an equivalent degree of conversion [6]. An added advantage of using microwaves was the possibility of incorporating an extraneous substrate with high microwave response to the adhesive components that subsequently became the heat source for the curing process. For instance, carbon nanotubes (CNTs) [8,9] and activated carbon powders [10,11] are such extraneous substrates that interact strongly with microwaves. In this regard, Rangari and coworkers [8] examined the microwave processing and characterization of the epoxy resin EPON 862/CNT nanocomposites in which curing the mixture with 2.45 GHz microwaves in a commercial microwave oven required only 10 min, rather than the 8 h typically required when curing by heating with a conventional oven. The effect of curing methods on the mechanical and morphological properties of carbon fiber epoxy composites was examined by Ma et al. [9], whose mechanistic analysis of the microwave curing process indicated that the resin at the surface layer and interior parts of the composites was cured with different forms, although the microwaves initiated no new chemical reactions during the curing process. In contrast, a Raman spectroscopic study by Soesatyo and coworkers [10] into the effects of microwave curing carbon-doped epoxy adhesive-polycarbonate joints showed that microwave processing changed the material’s characteristics. Additionally, experimental results by Li and coworkers [11] showed that drilling-induced de-lamination decreased, and the mode I interlaminar fracture toughness increased by more than 66% relative to traditional thermally cured samples. Although these earlier studies described interesting results, they were (in large part) conducted with commercial microwave ovens and industrial multimode applicators as the microwave heating devices. In this regard, temperature monitoring during microwave processing by Ma et al. [9] indicated an uneven electric field distribution in the domestic microwave oven used. Control of the microwave features is difficult at best with such devices, which precludes investigating details of the interactions between the microwaves and the substances involved in the curing process. In other words, although the curing phenomenon with microwaves is well established, it is not possible to ascertain the principles of adhesive curing by microwaves. 

Accordingly, the present study aimed at examining the curing characteristics of an epoxy adhesive using 2.45 GHz microwaves. A microwave apparatus was used capable of highly accurate microwave irradiation and precise temperature measurements during the curing process in comparison with a curing method that used ordinary conventional radiant heat or conduction heat transfer. The adhesive consisted of a combination of the highly versatile viscous epoxy resin bisphenol-A diglycidyl ether (BADGE), phthalic anhydride (PA) as the curing agent and 2-ethyl-4-methylimidazole (EM) as the curing accelerator. Both the curing agent and the curing accelerator are also highly versatile and storage-stable [7]. Thus, our objectives were two-fold: (i) elucidate the characteristics of curing with microwave heating and determine the raw material component that will be the driving force for microwave curing, and (ii) demonstrate whether the problems associated with adhesives can be overcome by taking advantage of the characteristics of microwaves.

## 2. Results and Discussion

### 2.1. Features and Advantages of Microwave Heating versus Conventional Heating on Curing an Adhesive

In the first approach, we examined the curing behavior of the adhesive using a commercially available microwave chemical synthesis system (Milestone General Co., Ltd., Flexi WAVE). After heating, the differences in adhesive curing between microwave heating and electric furnace heating were observed visually. The Milestone microwave device (see Section 3.3) was subsequently employed to observe the hardening of the adhesive in real time using the pre-existing hole at the top of the microwave device or else the pre-existing hole at the top of the electric furnace. 

**Figure 1:**
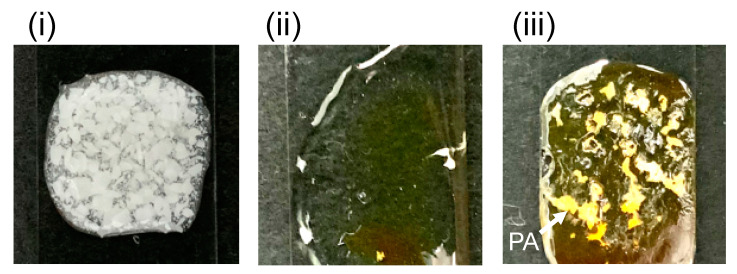
Photographs illustrating the curing of a (BADGE + PA + EM) {Bisphenol-A diglycidyl ether (BADGE), Phthalic anhydride (PA), 2-Ethyl-4-methylimidazole (EM)} adhesive before and after heating: (**i**) initial sample, (**ii**) curing the sample with microwaves for 10 min (microwave power, 500 W), and (**iii**) curing the sample in the electric furnace for 20 min.

The initial mix in which the white PA is dispersed in the unheated sample is illustrated in Figure 1i. To the extent that the curing agent is a solid white powder, the mixture with the transparent, albeit viscous epoxy resin, appeared cloudy initially; however, as the curing process evolved, the mixture became transparent so that the degree of curing was easily observed visually. After microwave heat-curing for 10 min, the (BADGE + PA + EM) adhesive mixture appeared as a pale-yellow transparent resin (Figure 1ii). Even after heating for 10 min in the electric furnace, the adhesive was sufficiently cured. However, visual observations clearly showed that unreacted PA was present near the center (Figure 1iii). That is, the advantage of microwave heating is that the components of the adhesive are guaranteed to react uniformly, in contrast to heating conventionally in the electric furnace that led to a non-uniform process. The radiant heat from the electric furnace cured only the surface of the adhesive near the heat source as the thermal conductivity of the adhesive is very low (0.3 W m K^−1^ as per the BADGE data sheet from the supplier), so that no heat penetrated into the inner parts of the resin. In other words, no thermosetting occurred in the inner parts. In contrast, with microwave heating, the incident microwaves penetrated sufficiently to the center to cause uniform thermosetting throughout. In addition, as the curing reaction is an exothermic process, the curing was accelerated by further self-heating of the adhesive via the microwaves (internal heating).

**Figure 2:**
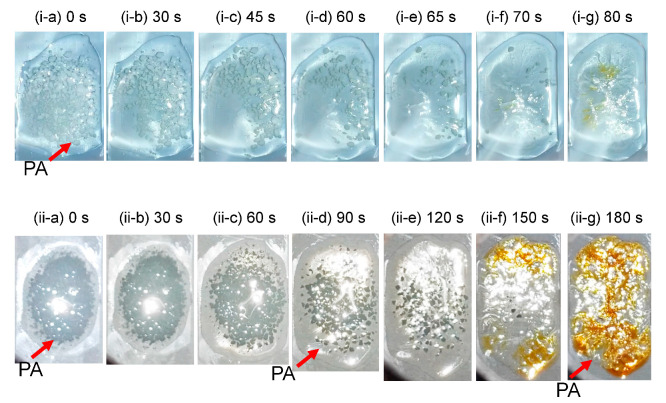
Sequential on-site photography of the curing of (BADGE + PA + EM) adhesives {Bisphe nol-A diglycidyl ether (BADGE), Phthalic anhydride (PA), 2-Ethyl-4-methylimidazole (EM)} (**i**) by microwave heating for times (**a**) through (**g**) of 0 to 80 s and (**ii**) by electric furnace heating for times (**a**) through (**g**) of 0 to 180 s.

In curing the (BADGE + PA + EM) adhesive mixture using microwave heating, the non-uniformly dispersed PA slowly began to flow toward the center from the lower left corner, as displayed in Figure 2(i-b), which contributed to the position of the electric field inside the waveguide. The adhesive sample inside the waveguide was installed at the bottom of the screen in Figure 2i; that is, at the maximal electric field density. The driving force in microwave heating required an electric field to affect the adhesive.

As illustrated from left to right in Figure 2i, the dispersed solid PA began to become transparent about 5 s after the start of microwave irradiation. Approximately one fourth of the PA was not observable from 45 to 60 s (Figure 2(i-c), (i-d)) of microwave irradiation. In fact, after 70 s, much of the PAs disappeared (Figure 2(i-f)), and after 80 s, the curing was complete (Figure 2(i-g)). The disappearance of PA, which appears white initially, progresses as the curing reaction evolves. Observing these series of recordings, we noted that the heating of the adhesive never occurred from the edge. In other words, heating appeared to proceed relatively evenly. It should be noted that in microwave irradiation in the single mode, the heating proceeds in a shorter time than in the multimode because in the former, the microwaves irradiate the adhesive more efficiently.

In comparison, to the extent that electrical power was used to pre-heat the (BADGE + PA + EM) mixture in advance when using an electric furnace, heating proceeded rapidly from the surface of the adhesive the moment the mixture was placed in the electric furnace. As shown in Figure 2(ii-b), the edge of the adhesive appears to be more cured 30 s after being introduced into the electric furnace. After this time, however, no further changes were observed for the white PA solid, and no PA flowed, contrary to what was observed under microwave heating. After 180 s (Figure 2(ii-g)), the surface seems to have hardened. In reality, however, the resin was not cured and was soft when pressed with a finger.

It is relevant to note that microwaves and electric furnaces apply heat differently to materials at the macroscale level.

### 2.2. Quality of Product

**Figure 3:**
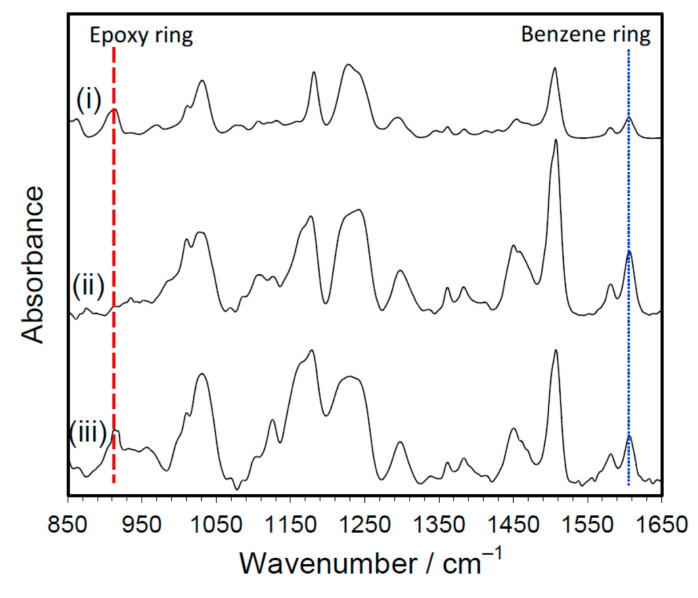
FT-IR spectra of the cured (BADGE + PA + EM) adhesive by microwave heating and by conventional heating in an electric furnace: (i) before curing, (ii) after microwave curing for 10 min, (iii) after electric furnace curing for 20 min.

The quality of the cured (BADGE + PA + EM) adhesive by microwave heating and electric furnace heating was examined by attenuated total reflectance FT-IR spectroscopy. Note that the sample used in the FT-IR analysis was the cured adhesive sample obtained in Figure 1. The intensity of the peak at 913 cm^−1^ (dashed red vertical line in Figure 3), attributed to the epoxy ring of BADGE [12,13,14,15,16], decreased with microwave curing. In contrast, the intensity of the peak at 1605 cm^−1^ (dotted blue vertical line in Figure 3) derived from the benzene ring present in BADGE [16] showed relatively no changes. No IR peak of the epoxy ring was seen in the cured (BADGE + PA + EM) adhesive subsequent to microwave heating for 10 min, which confirmed the occurrence of complete crosslinking of BADGE (Figure 3i) [12,13,14,15,16]. On the other hand, the peak of the epoxy ring was still visible after a 20 min curing period by electric furnace heating (Figure 3iii), indicating incomplete curing. Clearly, the curing process was uneven when comparing the two curing methods.

### 2.3. Power Consumption and Curing Time

The exact microwave power consumption in the curing of the (BADGE + PA + EM) adhesive was ascertained using the single-mode microwave device displayed in Section 3.3. The adhesive was introduced in a quartz tube that was subsequently located either at the position of maximal electric field (*E*-field) density or at the maximal magnetic field (*H*-field) position, and microwave power was 10 Watts. For comparison, an oil bath was used here for conventional heating as it was more efficient than heating with an electric furnace. As such, oil-bath heating provided a better means to compare the efficiencies between microwave heating and conventional heating.

Microwave heating occurs mostly by dielectric loss heating. In reality, however, microwave heating occurs through three heating mechanisms: (i) conduction loss heating, (ii) dielectric loss heating, and (iii) magnetic loss heating. In this regard, the thermal energy, *P*, produced per unit volume originating from microwave radiation can be estimated by Equation (1) [17]:

(1) $$P=\frac{1}{2}\sigma {\left|E\right|}^{2}+\pi f\varepsilon_{0}\varepsilon_{r}^{\prime \prime }{\left|E\right|}^{2}+\pi f\mu_{0}\mu_{r}^{\prime \prime }{\left|H\right|}^{2}$$

where |*E*| and |*H*| denote the strength of the microwaves’ electric and magnetic fields respectively, *σ* is the electrical conductivity, *f* is the frequency of the microwaves, *ε*_0_ is the permittivity in vacuum, *ε*_r_″ is the relative dielectric loss factor, *μ*_0_ is the magnetic permeability in vacuum, and *μ*_r_″ is the relative magnetic loss. 

The first term in Equation (1) expresses the conduction loss heating, the second term denotes dielectric loss heating, whereas magnetic loss heating is given by the third term. While microwave heating of the sample is governed mostly by dielectric loss heating, conduction loss heating involves mostly, but not exclusively (see below), solid materials. Therefore, microwave heating of materials is dictated by their electrical, dielectric, and magnetic properties. Moreover, the behavioral response of materials to microwaves changes whenever there is a change in microwave frequency. 

The characteristic features of microwave curing with respect to the microwaves’ electric field (*E*-field) and magnetic field (*H*-field) relative to oil-bath curing the (BADGE + PA + EM) mixture are reported in Table 1. Prior to the curing process, the initial temperature of the sample was 25 °C, following which the times needed to reach the curing temperature of 140 °C were determined. Irradiation with the microwaves’ electric field caused the curing time of (BADGE + PA + EM) to be shortened (32 s) considerably, especially with 20 W microwaves, while the temperature did not rise above 67 °C on irradiation with the microwaves’ magnetic field, even when using 20 Watt microwaves. By comparison, it took 80 s to reach 140 °C in the curing of the (BADGE + PA + EM) adhesive with the pre-heated oil bath, albeit at a much greater average power consumption (129 Watts, nearly 16 times greater) than the equivalent 8 Watts with the microwaves for the same time of 80 s. In addition, curing the adhesive with the oil bath revealed that the PA component remained in the center of the sample, thereby yielding an uneven curing of the adhesive—see, for example, Figure 1iii.

### 2.4. Mechanism of Microwave-Assisted Heating in Curing the Adhesive

Interactions between microwaves and the three raw materials (BADGE, PA, and EM) separately or as a mixture were investigated from a heating experiment that involved the single-mode microwave equipment (see Section 3.3) and a Network analyzer to determine the relevant dielectric parameters. 

**Figure 4:**
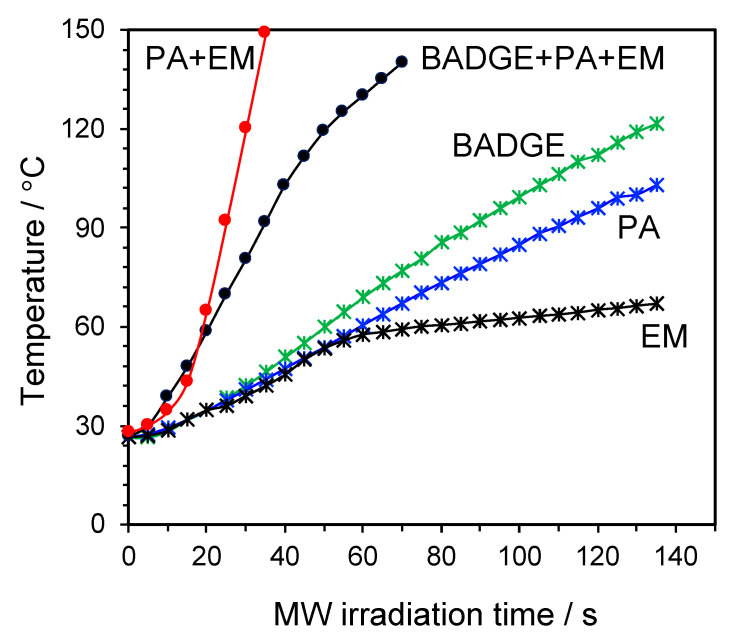
Temperature changes resulting from microwave irradiation by the electric field (*E*-field; 10 W) using a single-mode applicator and a semiconductor microwave generator for each of the components (BADGE, PA, EM) and for the mixtures (BADGE + PA + EM) and (PA + EM).

The effect that microwave heating had on each of the components (BADGE, PA, and EM) and on the mixtures (BADGE + PA + EM) and (PA + EM) was examined by placing a sample in a quartz tube followed by heating with the microwaves’ *E*-field (10 Watts) while observing the temperature changes. For each of the components (BADGE, PA, and EM), the temperature increase tended to be somewhat similar, as reported in Figure 4. After 60 s of irradiation with microwaves, the temperature rise for EM began to decrease toward a plateau. At room temperature, naked EM is a clay-type solid that turns into a liquid at temperatures above 54 °C (melting point). The decrease in the temperature rise near the melting point of EM suggests that the efficiency of microwave heating depends on the state of matter. In this regard, the microwave heating efficiency is governed by the dielectric properties of materials, as reported in the earlier study by Sumi and coworkers [18], who indicated that there exists a relationship between solution viscosity and microwave heating, consistent with the similar tendency displayed herein by the EM component. 

Significant enhancement of temperature rise proceeded with initial irradiation with microwaves. Therefore, it seems that microwave heating promotes the chemical reaction between the curing agent and the plasticizer. To further separate the factors that influence the heating rate, the mixture of PA and EM ((PA + EM) in Figure 4) was microwave-heated, which led the heating efficiency to further improve after 20 s. The temperature reached 140 °C after about 74 s for the three-component mixture. Additionally, the mixture (PA + EM) displayed a higher microwave heating rate than did the (BADGE + PA+ EM) mixture. Moreover, the heating rate was more than 3 times better than those of PA or EM alone.

**Scheme 1:**
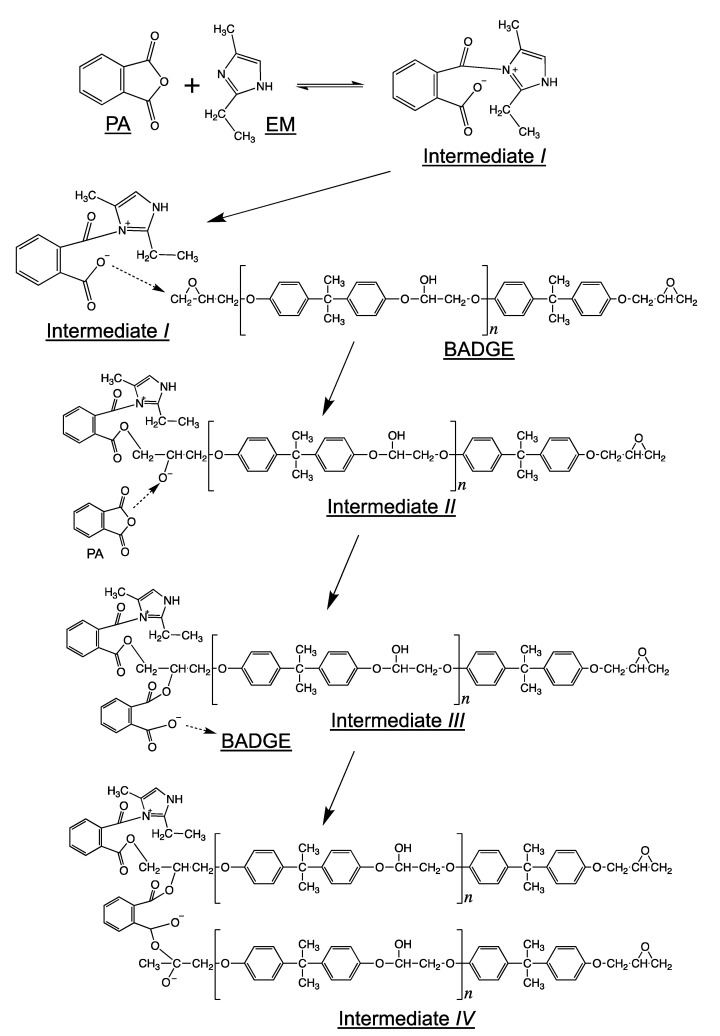
Inferred synthetic reaction of the crosslinking and curing of the three-component adhesive: Bisphenol-A diglycidyl ether (BADGE), Phthalic anhydride (PA), and 2-Ethyl-4-methylimidazole (EM). Summarized from the results of Onizuka et al. [19].

A discussion on microwave curing of adhesives cannot be undertaken simply on the basis of the microwave heating efficiency of each raw material, as microwave heating in the curing of the adhesive seems to vary greatly depending on the step of the curing reaction. In this regard, the study by Onizuka [19] is of particular interest as it suggested a reaction mode by which the curing mechanism of the (BADGE + PA + EM) mixture might occur (Scheme 1). The basis of this curing mechanism allows for a consideration as to where the microwaves interact with the mixture components.

Initially, the curing agent (PA) reacts with the curing accelerator (EM) at low temperature to form the charged intermediate ***I***, subsequent to which the C–O^−^ group attacks the epoxy ring of BADGE to form intermediate ***II***. Another PA attack on the C–O^−^ group of intermediate ***II*** produces intermediate ***III***, subsequent to which addition of another BADGE to the C–O^−^ group of intermediate ***III*** repeatedly leads to additional crosslinking to yield intermediate ***IV*** and ultimately to the curing of the adhesive.

The element that governs the heating by the microwave *E*-field is the dielectric loss (*ε*″), which consists of two terms: dielectric heating (first term in Equation (2)) and Joule heating (second term in Equation (2)) [4]:

(2) $$\varepsilon^{\prime \prime }=\frac{\varepsilon_{s}-\varepsilon_{\infty }}{1+\omega^{2}\tau^{2}}\omega \tau +\frac{\sigma }{\omega }$$

where *ε*_s_ is the dielectric constant at low frequencies and *ε*_∞_ is the dielectric constant at high frequencies, *ω* is the angular frequency of the electromagnetic radiation, *σ* is the ionic conductivity of the electrolyte solution, and *τ* is the relaxation time taken as a measure of the time required for water to rotate; that is, *τ* = 4*π* *η* *r*^3^/*κT*, where *r* is the molecular radius, *κ* is Boltzmann’s constant, *T* is the Kelvin temperature, and *η* is the viscosity that may be considered to cause the delay of molecules (or particles) to respond to the field changes.

**Figure 5:**
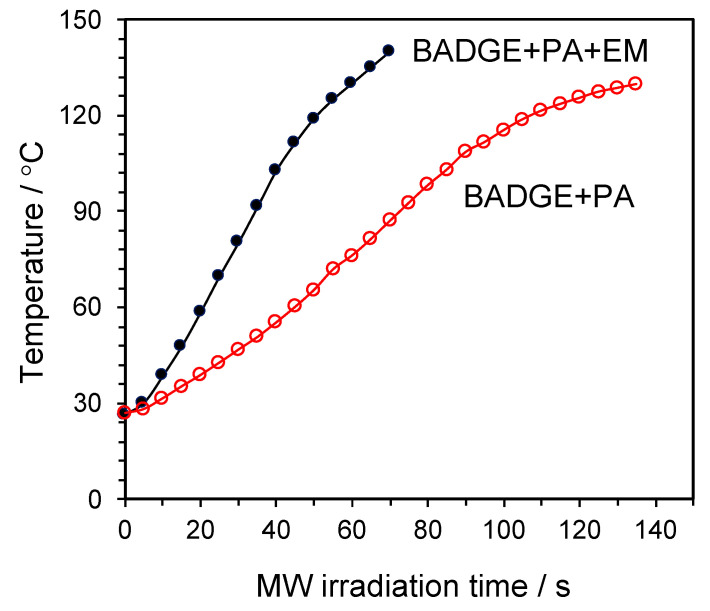
Temperature change due to electric field (*E*-field) heating with 10 W microwaves using a single-mode applicator and a semiconductor microwave generator for the (BADGE + PA + EM) and (BADGE + PA) samples.

Although each of the components of the adhesive is a somewhat poor absorber of the microwave energy, they nonetheless cause some microwave heating. The intermediates produced by the crosslinking reactions, however, do induce microwave heating and because of their charged nature, dielectric heating and Joule heating run parallel to generate heat. Accordingly, microwave heating was performed without the curing accelerator EM; that is, microwave heating was carried out on the (BADGE + PA) mixture. Onizuka [19] has shown that the external crosslinking reaction between BADGE and the curing agent PA can occur even without the curing accelerator EM. Though intermediate ***I*** is not generated, the crosslinking rate is therefore expected to be slower, with some repercussions on the heating efficiency of the microwaves as the formation of intermediates will also be slower. This expectation was confirmed by the results illustrated in Figure 5, in which the temperature increase for the (BADGE + PA) mixture was significantly slower than for the (BADGE + PA + EM) mixture.

### 2.5. Relationship between the State of the Curing Agent (Liquid or Solid) and the Heating Rate

The changes of state of matter (from solid to liquid) alter the microwave heating efficiency, as seen with pure EM heating (Figure 4). It should be noted here that PA at room temperature is a solid and is observed as a white powder at 131 °C, which is above the boiling point, or if the chemical reaction with BADGE or EM were not to proceed. Therefore, we investigated whether the reaction promotion of (PA + EM) in Figure 4 was due to the intermediates generated (intermediates ***I***–***IV*** in Scheme 1) by the curing reaction or by the change of state of PA from a solid to a liquid. As PA is highly viscous at room temperature, we substituted PA with the low-viscosity liquid curing agent tetra- propenyl succinic anhydride (TA; C_16_H_26_O_3_) for the microwave curing process. In the curing mechanism with TA, the reaction between the ring-opened five-membered ring and EM goes through an ionic intermediate, similar to the curing mechanism of PA (Scheme 1). Therefore, the difference in heating rate is rather obvious in this experiment and depends on whether the curing agent is a solid (PA) or a liquid (TA).

**Figure 6:**
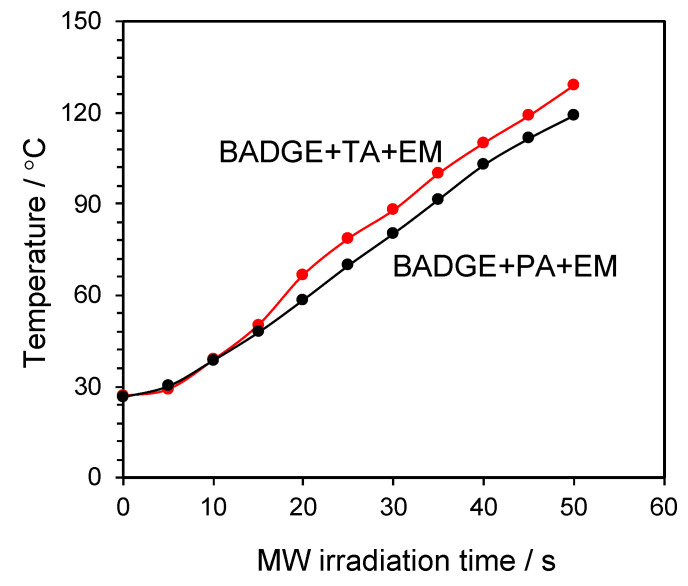
Temperature changes of naked (BADGE + TA + EM) versus (BADGE + PA + EM) adhesives under microwave irradiation by the electric field (*E*-field; 10 W) from a single-mode applicator and a semiconductor microwave generator.

Comparing the temperature increases of (BADGE + PA + EM) versus (BADGE + TA + EM) revealed no large differences in the rates of temperature increases (Figure 6), which infers that the difference in the state (solid or liquid) of the curing agent makes no difference in the curing rate when microwaves are implicated.

### 2.6. Understanding the Microwave Effect of Dielectric Loss

How microwaves interact with the constituent molecules in the adhesive from the experimental results of microwave heating was confirmed by measuring the relative dielectric loss, *ε*_r_″, of each of the constituents using the Network analyzer. Results are reported in Table 2. Note that the relative dielectric factor (*ε*_r_′) data is also shown for reference. To the extent that the relative dielectric loss changes with temperature, it was measured at room temperature (25 °C), as well as at 60 and 100 °C. The relative dielectric loss of ion-exchanged water was measured for index purposes. The relative dielectric loss factors of each of the constituents and mixtures at ambient temperature were significantly lower than that of water, whose dielectric loss decreased with increase of temperature, while the relative loss of each of the constituents and mixtures increased as the temperature increased. It was also shown that each substrate (BADGE, PA, and EM) is significantly less efficient in microwave heating than is water, consistent with the results in Figure 4. Those of PA and EM were even lower than that of the BADGE constituent. Comparing the (BADGE + PA) and (BADGE + EM) mixtures, the former (BADGE + PA) mixture shows improved microwave heating efficiency. On the other hand, the (BADGE + EM) mixture shows no improvement of the heating efficiency. Therefore, it appears that PA is the factor that determines the enhancement of the heating efficiency.

In the previous section, we speculated that the reason for the rapid heating of (PA + EM) shown in Figure 4 was due to the formation of a series of intermediates by the curing reaction. The value of the dielectric loss of (PA + EM) at 25 °C does not show a significantly high value. However, at 60 °C, the *ε*_r_″ was 6.836, a value 14 times greater than at 25 °C. Furthermore, the value at 100 °C was 17-fold greater (*ε*_r_″ = 8.432). Evidently, the reaction will improve by the heat generation efficiency of the microwaves.

The sample consisting of (BADGE + PA + EM) showed a far greater dielectric loss, *ε*_r_″, than for each of the constituents, and it also improved at the higher temperatures. Next, in order to clarify that the microwave heating rate is also present in the reaction intermediate in addition to the interaction between the materials and the microwaves, the dielectric loss of the cured sample was also measured. Subsequent to curing the three-component adhesive by microwave heating, it was cooled to room temperature and the relative dielectric loss was measured, which turned out to be significantly low. This suggests that microwave heating no longer impacted the adhesive after the curing process, and that the rate-determining factor of microwave heating was the chemical reaction.

### 2.7. Protection of Thermal Deterioration of the Base Substrate

**Figure 7:**
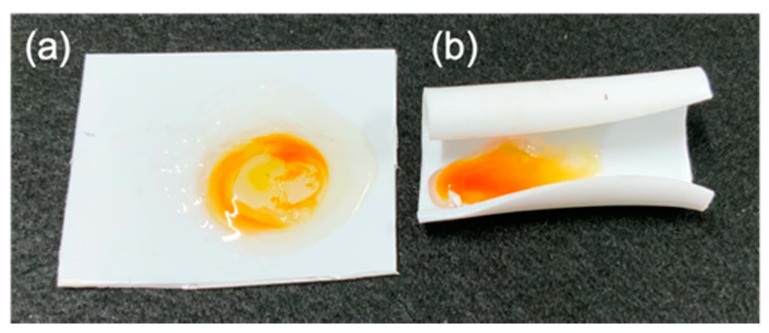
Photographs illustrating the curing of the (BADGE + PA + EM) adhesive on a polystyrene plate by (**a**) microwave heating (500 W, 20 min) and (**b**) by heating in an electric furnace (20 min).

From the curing sample data of Table 2, it is expected that the cured adhesive will hardly generate heat from the microwaves. This feature of heating may protect against deterioration of the substrate, which is a disadvantage of thermosetting adhesives. A cured sample data in Table 2 shows that even if the cured adhesive were continuously irradiated with microwaves, further heat generation could be prevented, and the base substrate would not be exposed to heat for a long time, so that deterioration can be suppressed. Therefore, to substantiate this hypothesis, demonstration of curing was conducted by placing a sample of the (BADGE + PA + EM) adhesive onto two polystyrene plates, which were subsequently heated either by the microwaves or else in the electric furnace. Note that polystyrene, which has low microwave heat generation efficiency, was used as the base substrate. The microwave device used in this experiment was the commercially available Milestone multimode system. The curing of the (BADGE + PA + EM) adhesive was complete after about 6 min of microwave irradiation. The continued heating for 20 min led only to a slight temperature increase of the polystyrene plate (Figure 7a). On the other hand, when the polystyrene plate was heated in an electric furnace, it began to deform within about 5 min with continued heating, causing the plate to deform completely (Figure 7b). Clearly, unlike curing by electric furnace heating for 20 min that caused significant thermal deterioration of the base material, microwave curing was rapid, gave a high-quality product, and was selective as microwave heating affected only the adhesive mixture. The same experiment was conducted in the single-mode system (see Section 3.3); again, no distortion of the polystyrene plate was observed.

The penetration depth of the microwaves (*D_p_*) into a substance can be calculated from the relative permittivity and the relative dielectric loss factors using Equation (3) [20], with both parameters being measured with a Network analyzer. The penetration depth, *D_p_* (in cgs (centimeter-gram-second) units), refers to the depth at which microwaves pervade into the material and to the power flux that has fallen to 1/*e* (=0.368) of its surface value. That is, it denotes the depth at which the power density of the microwaves is reduced to 1/*e* of its initial value.

(3) $$D_{p}=\frac{\lambda }{4\pi }{\left[\frac{2}{\varepsilon^{\prime }\left(\sqrt{1+{\left(\varepsilon^{\prime \prime }/\varepsilon^{\prime }\right)}^{2}}-1\right)}\right]}^{1/2}$$

where *λ* is the wavelength of the microwave radiation (*λ*_(2.45GHz)_ = 12.24 cm). 

The permeation depth before curing the (BADGE + PA + EM) mixture was 5.1 cm at 25 °C, while the permeation depth was 21.5 cm after curing the (BADGE + PA + EM) adhesive. It is clear that the cured adhesive is permeable to microwaves. 

## 3. Materials and Methods

### 3.1. Chemical Components of the Adhesive

**Scheme 2:**
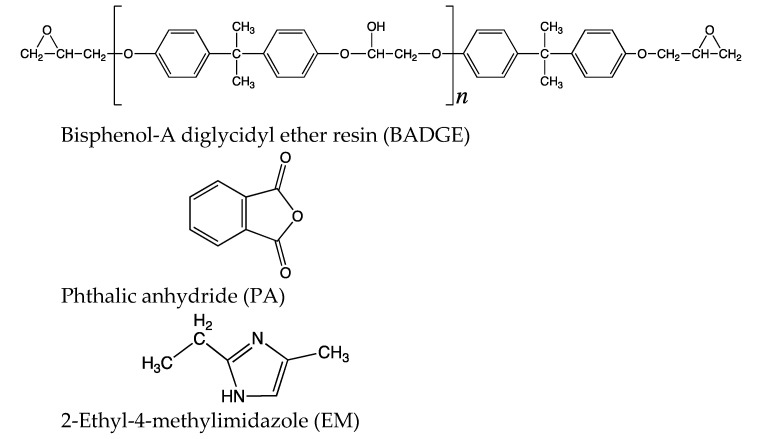
Chemical formulae of Bisphenol-A diglycidyl ether resin (BADGE), Phthalic anhydride (PA), and 2-Ethyl-4-methylimidazole (EM).

The colorless viscous bisphenol-A diglycidyl ether (BADGE; JER827, Mitsubishi Chemical Co., Tokyo, Japan) was the epoxy resin, the white solid phthalic anhydride (PA; C_8_H_4_O_3_; Tokyo Chemical Industry Co., Ltd., Tokyo, Japan) was the curing agent, while the 2-ethyl-4-methylimidazole liquid (EM; C_6_H_10_N_2_; Tokyo Chemical Industry Co., Ltd.) was the curing accelerator (see Scheme 2).

The adhesive mixture of the three components is abbreviated as (BADGE + PA + EM), and its curing temperature was 123.4 °C measured by Differential Scanning Calorimetry (DSC). As an experimental condition, the temperature for heating by the devices was set at 140.0 °C. 

### 3.2. Curing by Microwaves and by Conventional Heat 

To compare the characteristics of adhesive curing by microwaves, experiments were conducted in a commercially available microwave chemical synthesis system (Milestone General Co., Ltd., Flexi WAVE) equipped with a multimode applicator and a mode stirrer. This microwave device was used to obtain the experimental data portrayed in Figure 1, Figure 2 and Figure 3. To compare with the result of this microwave heating, curing by conventional heat transfer was achieved, unless noted otherwise, using the radiant heat from an electric furnace (model VTDS-16K; Isuzu Motors Ltd., Tokyo, Japan). Exactly 2.00 g of BADGE, 1.60 g of PA, and 0.20 g of EM were mixed in a glass container, from which a 0.50 g sample was subsequently placed onto a quartz glass slide (400 mm × 15 mm × 1 mm to cover an area of ca. 400 mm^2^). Note that this mixing ratio was based on information from the Mitsubishi Chemical Co. that provided the BADGE resin. The electric furnace was preheated to an internal temperature of 150 °C prior to introducing the sample into the hot furnace. All experiments were repeated three times under otherwise identical conditions, and there were no differences between the experiments.

### 3.3. Microwave-Assisted Curing with Controlled Microwaves

**Figure 8:**
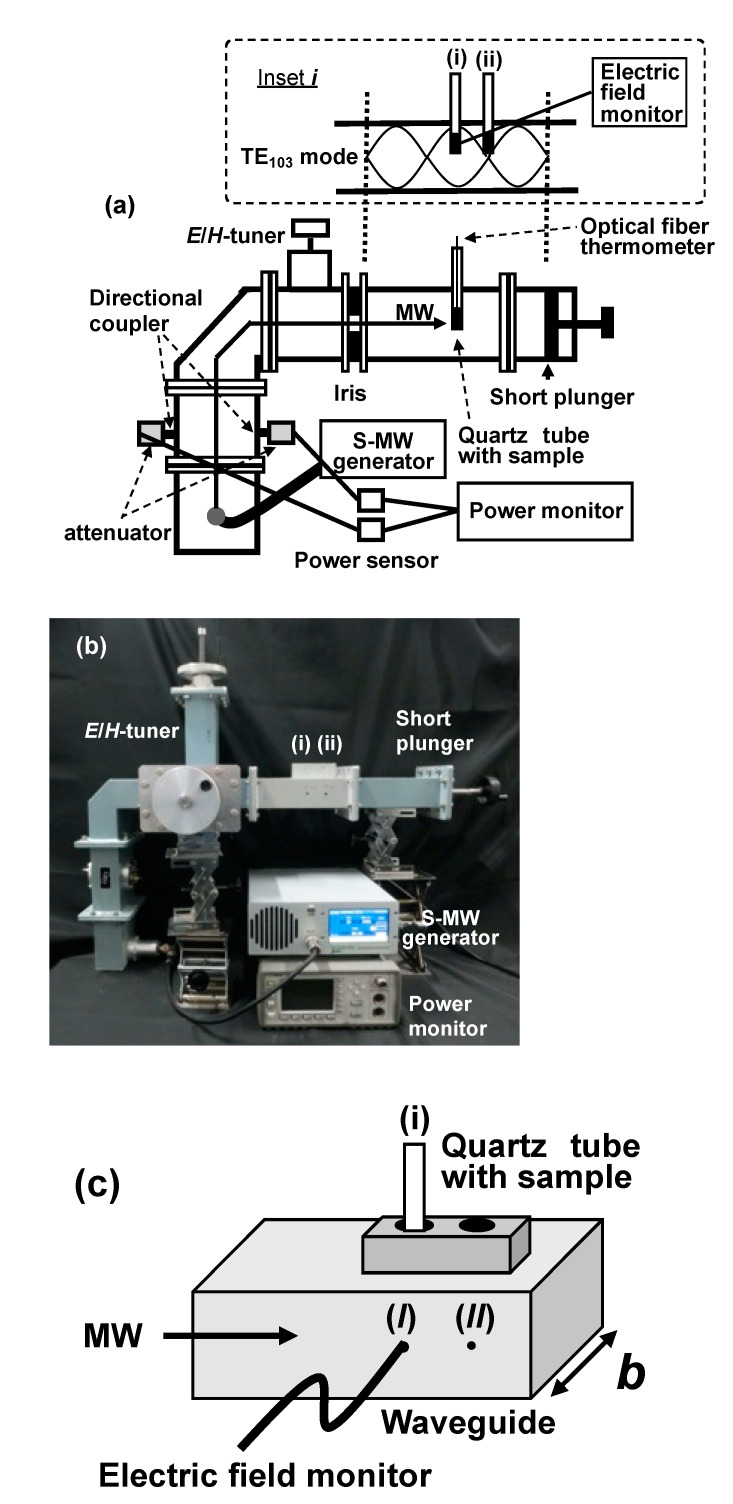
(**a**) Schematic and (**b**) photograph of the microwave irradiation system consisting of a 2.45 GHz semiconductor microwave generator (S-MW generator), an optical fiber thermometer, a power sensor, a power monitor and a single-mode applicator. Inset *i* in (**a**): distribution of the (i) electric field inside the single-mode applicator and installation position of the sample. (**c**) Image of the sample introduced at the maximum electric field position (***I***) of the single-mode applicator (waveguide). The width *b* denotes the *b* in Equation (3).

The interaction(s) between the microwaves and the adhesive components was examined using a microwave heating device consisting of a single-mode applicator connected to a semiconductor microwave generator. This device afforded highly accurate microwave irradiation control (Figure 8). The microwave irradiation setup with the single-mode cavity TE_103_ (transverse electric 103 mode), used to irradiate the reactor contents and schematically illustrated in Figure 8, included a short plunger, an iris, and an *E*/*H*-tuner. The continuous microwave radiation was generated from the 2.45 GHz microwave semiconductor generator containing an isolator (LEANFA Srl; GEAP-A003A01; 2.45 GHz; maximum power, 250 Watts). The length of the single-mode applicator is 49 cm, but moving the short plunger actually changes the length of the cavity. The resonance of the microwaves was adjusted with the iris and the plunger at 1.5 cycles. Heating the sample solution was achieved by positioning the quartz tube in the single-mode microwave apparatus of Figure 8a,b, either at positions of maximal electric field density (*E*-field; position (i) in Figure 8a, inset *i*) or at the maximal magnetic field density (*H*-field; position (ii) in Figure 8a, inset *i*) within the waveguide.

The wavelength of propagation of the microwaves in the TE_103_ mode within the waveguide was ca. 14.78 cm, estimated from Equation (4) [21]:

(4) $$\lambda =\frac{\lambda_{0}}{\sqrt{1-{\left(\lambda_{0}/2b\right)}^{2}}}$$

where *λ* is the wavelength in the waveguide, *λ*_0_ (2.45 GHz) = 12.24 cm is the wavelength in vacuum given by *c*/*f* (with *c* being the speed of light, 2.9979 × 10^10^ cm s^−1^, and *f* being the microwave frequency 2.45 × 10^9^ s^−1^, i.e., 2.45 GHz), and *b* is the width of the waveguide, 10.92 cm (Figure 8c). The maximal position of the *E*-field from the iris was at 1/2 the wavelength of the standing wave in the waveguide, namely 7.39 cm. The maximal position of the *H*-field from the iris was located at ¾ of the wavelength of the standing wave in the waveguide, namely 11.09 cm [22]. An electric field monitor with a probe (Fuji Electronic Industrial Co., Ltd., Tsurugashima, Japan, J-213 *E*-monitor) was used to maintain the sample at the maximal position of the *E*-field density, as the reproducibility of such experiments is often diminished if such operations are neglected. The density was measured from a 2 mm hole (*I*) on the side of the waveguide (Figure 8c). On the other hand, if the electric field at hole (*II*) were near zero, then the magnetic field would be at its maximum. Note that the reflected power determined experimentally was less than 0.1 W.

The incident and reflected waves of the microwave were measured precisely with a power meter (Keysight technologies, Santa Rosa, CA, USA, E4419B EPM series dual-channel power meter) and a power sensor (Keysight technologies, E9300A E-series average power sensor), respectively. These were cabled in the directional coupler (Fuji Electronic Industrial Co., Ltd., Tsurugashima Saitama, Japan E-049 directional coupler) on the waveguide by inserting an attenuator (Keysight technologies, Santa Rosa, CA, USA, 8496B manual attenuator) between the directional coupler and the power sensor. The actual microwave output was attenuated and displayed on the power meter. Calibrations were performed by Fuji Electronic Industrial Co., Ltd. Temperatures of the solutions were measured at 3 s intervals with an optical fiber thermometer (FL-2000, Anritsu Meter, Co., Ltd., Tokyo, Japan). Appropriate mixed samples of (BADGE + PA + EM) consisted of 0.50 g of BADGE, 0.40 g of PA, and 0.05 g of EM. Subsequently, a 0.95 g sample of the mixture was placed in a quartz tube (inner diameter, 1.0 mm; outer diameter, 1.2 mm; height, 120.0 mm). For comparison with microwave heating (Figure 8), a test tube containing a sample of the adhesive mixture was also immersed in an oil bath preheated to 150 °C for a rapid and uniform heating of the sample. 

The difference in structure of the (BADGE + PA + EM) adhesive between microwave heating and conventional heating after curing was investigated by the attenuated total reflection (ATR) technique using a diamond prism in the FT/IR-4600 spectrophotometer (JASCO Corporation). Differential scanning calorimetry was performed using a Hitachi High-Tech X-DSC7000 apparatus (sample, about 15 mg; heating rate, 10 °C min^−1^). The relative dielectric loss factor (*ε*_r_″) and relative dielectric factor (*ε*_r_′) of the components and the various mixtures were determined at temperatures of 25, 60, and 100 °C using, respectively, an Agilent Technologies HP-85070B Network Analyzer and an Agilent dielectric high-temperature probe (measures up to ~200 °C). Powder or liquid samples were measured by placing a sufficient amount of a sample in a quartz petri dish (diameter, 2 cm) and pressed against the probe. In contrast, the solid sample was measured by flattening the surface and pushed against the probe. The experiment was repeated with three different samples, and no differences were observed in the results.

## 4. Concluding Remarks

This study has confirmed the rapid, high-quality, and selective curing characteristics of microwave heating in the curing of an epoxy adhesive. The formation of charged intermediates generated by the crosslinking reaction also induced the generation of microwave heating through the Joule effect. On the other hand, the cured adhesive does not generate heat due to microwaves, so it can be combined with a substrate that does not absorb microwaves to prevent deterioration of the substrate. This solves the problem of thermosetting raw adhesives. By using a small sample in this experiment, we were able to understand the detailed interaction between the microwave heating and epoxy adhesive curing. With this as a basic factor, and the use of a larger microwave device that combines a semiconductor generator and antenna technology [23], it should be possible to cure larger samples to yield high-precision adhesives.

## Figures and Tables

**Table 1:** Microwave power and curing times for the (BADGE + PA + EM) adhesive using the microwaves’ electric field heating (*E*-field), the microwaves’ magnetic field heating (*H*-field), and oil-bath heating to reach a temperature of 140 °C.

Heating Condition	Microwave Power (Watt)	Time(s) to Reach a Curing Temperature of 140 °C
*E*-field	5	150
*E*-field	10	74
*E*-field	15	49
*E*-field	20	32
*H*-field	20	Not reached (maximal T reached: 47 °C)
Oil bath	129 ^a^	81

^a^ Electrical power consumption.

**Table 2:** Relative dielectric loss (*ε*_r_″) and relative dielectric factor (*ε*_r_′) of each component of the adhesive (BADGE + PA + EM) mixture and other mixtures, together with that of ion-exchanged water (H_2_O).

System	25 °C		60 °C		100 °C	
	*ε*_r_″	*ε*_r_′	*ε*_r_″	*ε*_r_ ′	*ε*_r_″	*ε*_r_ ′
H_2_O	8.259	75.81	4.334	68.78	2.701 *	62.75
BADGE	0.398	2.647	0.399	3.095	0.796	4.003
PA	0.190	1.492	0.217	1.542	0.457	1.281
EM	0.176	2.693	0.204	3.130	0.218	2.751
(BADGE + PA)	0.042	1.263	0.296	3.284	0.999	9.122
(BADGE + EM)	0.259	3.110	0.286	3.131	0.360	3.121
(PA + EM)	0.495	10.28	6.836	11.58	8.432	12.06
(BADGE + PA + EM)	0.745	3.524	1.892	3.947	2.647	6.184
Cured sample	0.014	5.210	0.014	5.481	0.015	5.881

* Measured at 90 °C instead of 100 °C for ion-exchanged water.

## Data Availability

The data presented in this study are available on request from the corresponding author.

## Sample Availability

Samples of the compounds are not available from the authors.
